# Real-Time Leaflet Monitoring Using Intravascular Ultrasound During Transcatheter Aortic Valve Implantation

**DOI:** 10.1016/j.jaccas.2025.104168

**Published:** 2025-07-16

**Authors:** Tomohiro Suenaga, Kenichi Ishizu, Akihiro Isotani, Shinichi Shirai, Kenji Ando

**Affiliations:** Department of Cardiology, Kokura Memorial Hospital, Kitakyushu, Japan

**Keywords:** coronary obstruction, intravascular ultrasound, TAVI

## Abstract

Coronary obstruction (CO) is a rare but fatal complication after transcatheter aortic valve implantation. “Prophylactic” chimney stenting is effective in patients with high CO risk; however, concerns about future coronary access and stent thrombosis have emerged. We experienced a case in which intravascular ultrasound enabled real-time monitoring of displaced native leaflets during a self-expandable transcatheter heart valve expansion, consequently avoiding chimney stenting. This case highlights the importance of intravascular ultrasound to accurately assess CO during self-expandable transcatheter aortic valve implantation and prevent unnecessary chimney stenting.

## Graphical Abstract

**Figure:**
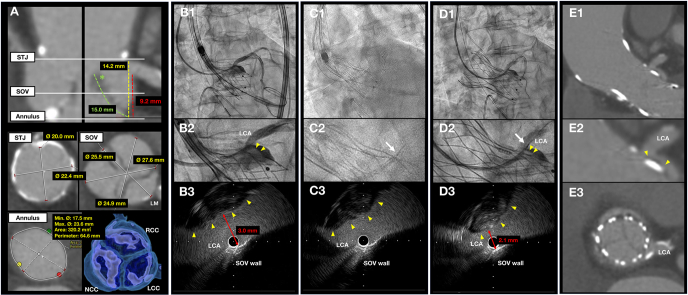

**Take-Home Message**

• This case highlights the importance of intravascular ultrasound to accurately assess coronary obstruction (CO) risk during complex transcatheter aortic valve implantation with a high CO risk.

A 75-year-old woman with a symptomatic severe aortic stenosis was referred for transcatheter aortic valve implantation (TAVI). Preprocedural computed tomography revealed a low left coronary artery (LCA) height (9.2 mm) with shallow Valsalva (24.9 mm) and a long thickened leaflet (15.0 mm) at the left coronary cusp (Figure 1A), indicating a high risk for coronary obstruction (CO).

**Figure 1:**
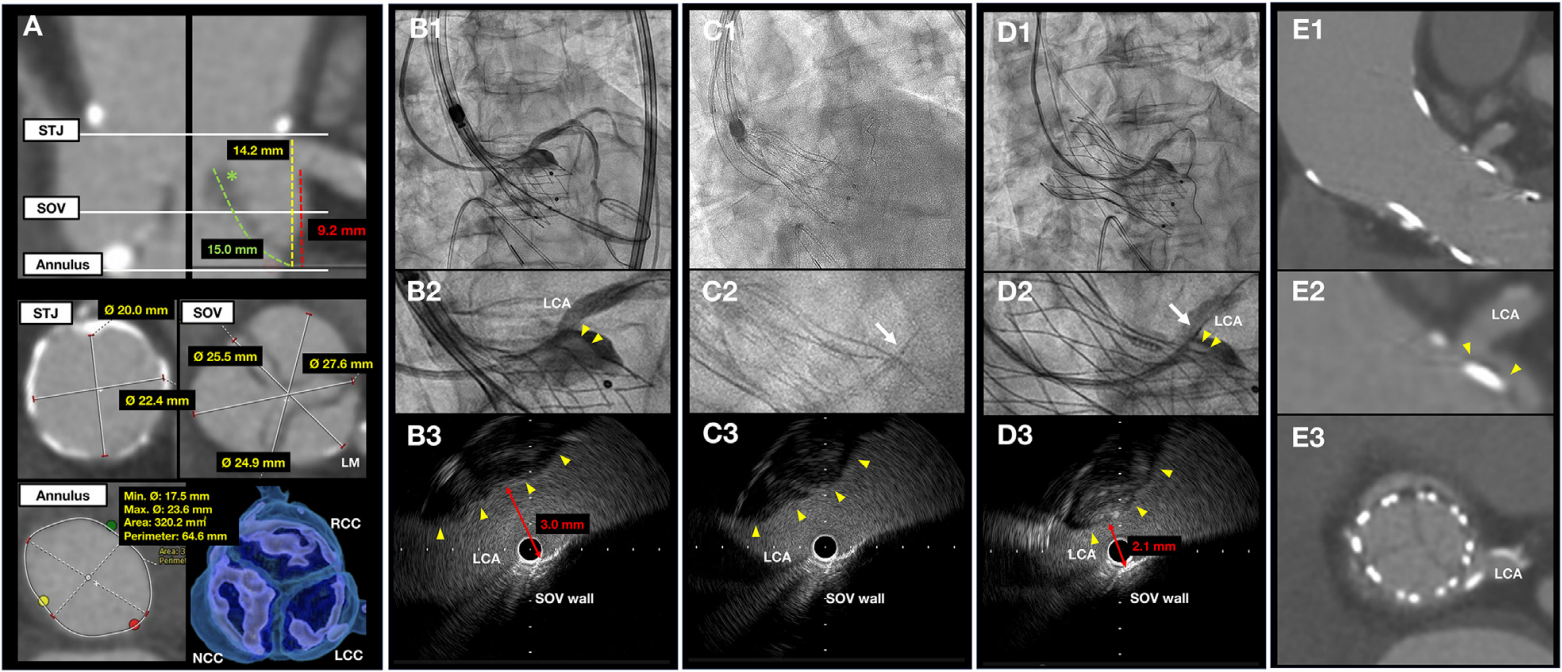
Real-Time Leaflet Monitoring Using IVUS During Transcatheter Aortic Valve Implantation (A) Preprocedural computed tomography showing a long LCC leaflet (green asterisk) and low LCA height. (B) At the point of no recapture, (C) during the THV release, and (D) after the THV release, IVUS images enabled clear visualization of a positional relationship between the tilted-up leaflet (yellow arrowheads) and the LCA ostium. White arrows indicate IVUS catheter placement in the LCA ostium. (E) Postprocedural computed tomography showing a patent LCA ostium. IVUS = intravascular ultrasound; LCA = left coronary artery; LCC = left coronary cusp; NCC = noncoronary cusp; RCC = right coronary cusp; SOV = sinus of Valsalva; STJ = sinotubular junction; THV = transcatheter heart valve.

Given her high surgical risk, the heart team opted to perform TAVI with LCA protection. After the LCA was wired through the radial approach and an intravascular ultrasound (IVUS) catheter was placed in the LCA ostium with the support of a guide extension catheter, we expanded a 23-mm valve (Navitor; Abbott). Angiography at the point of no recapture showed the shifting of native leaflets toward the LCA ostium, whereas IVUS revealed a leaflet-to-coronary distance of 3.0 mm (Figure 1B). We therefore decided to fully expand the prosthesis very slowly with the real-time risk assessment of CO using IVUS. Chimney stenting was planned only if IVUS confirmed significant stenosis of the LCA ostium during prosthesis expansion (Figure 1C, Video 1). While the transcatheter heart valve was fully expanded, IVUS showed that the tilted-up leaflet was gradually approaching the LCA ostium, but the leaflet-to-coronary distance eventually only narrowed to 2.1 mm. Preserved coronary blood flow was confirmed, and the procedure was completed without stenting (Figure 1D, Video 1). Postprocedural computed tomography also confirmed a patent LCA ostium (Figure 1E).

CO is a rare but fatal complication after TAVI.^1^ “Prophylactic” chimney stenting is effective in high-risk cases; however, there are emerging concerns regarding future coronary access and the risk of stent thrombosis. Real-time IVUS assessment while expanding self-expandable valves may accurately assess CO risk and potentially prevent unnecessary chimney stenting. From a long-term perspective, avoiding unnecessary chimney stenting not only reduces the subsequent excess bleeding risk associated with multiple antithrombotic agents but also provides favorable future coronary access, which would be particularly important in patients requiring repeat intervention. Furthermore, “bailout” chimney stenting can be delivered via the guide extension catheter immediately if IVUS confirmed a coronary artery ostium stenosis during the prosthesis expansion. However, it should be noted that some proficiency in IVUS image evaluation is required for this technique and that IVUS has certain constraints on its resolution and imaging depth.^2^

## Funding Support and Author Disclosures

Dr Ishizu is the proctor of intracardiac echocardiography–guided TAVI for Johnson and Johnson. Dr Shirai is the proctor of transfemoral TAVI for Edwards Lifesciences, Medtronic, and Abbott Medical. All other authors have reported that they have no relationships relevant to the contents of this paper to disclose.

## Appendix

Video 1 Fluoroscopic and Intravascular Ultrasound Video During Prosthesis Release
